# Supplementary material for: Cervical HPV Infections, Sexually Transmitted Bacterial Pathogens and Cytology Findings—A Molecular Epidemiology Study
Source: Pathogens. 2023 Nov 14;12(11):1347. doi: 10.3390/pathogens12111347 (PMC10675441; doi:10.3390/pathogens12111347)
Supplement: Supplementary file 1 [file pathogens-12-01347-s001.zip › pathogens-2634054-supplementary.pdf]

Supplement

Table S1: Distribution of bacterial STIs (other than HPV) expression in relation to HPV positivity (any type, low risk, high risk or concurrent low/high risk genotypes).

STIs	Total	HPV (+) n=351 (25.6%)	HPV (-) n=1020 (74.40%)	OR (95% CI) (p)	HPV HR (+) n=293 (21.37%)	HPV HR (-) n=1078 (78.63%)	OR (95% CI) (p)	HPV LR (+) n=100 (7.29%)	HPV LR (-) n=1271 (92.71%)	OR (95% CI) (p)	HPV Both HR & LR (+) n=42 (3.06%)	HPV Both HR & LR (-) n=1329 (96.94%)	OR (95% CI) (p)
STIs (+) n (%)	460 (33.55)	178 (38.7)	282 (61.30)	**2.69 (2.10-3.46) (<0.05)**	152 (33.04)	308 (66.96)	**2.70 (2.07-3.51) (<0.05)**	54 (11.74)	406 (88.26)	**2.50 (1.66-3.77) (<0.05)**	28 (6.09)	432 (93.91)	**4.15 (2.16-7.97) (<0.05)**
STIs (-) n (%)	911 (66.45)	173 (18.99)	738 (81.01)		141 (48.12)	770 (84.52)		46 (5.05)	865 (94.95)		14 (1.54)	897 (98.46)	
	RR	2.04 (1.71-2.43)	0.76 (0.70-0.82)	**x^2 p<.0001**	2.13 (1.75-2.61)	0.79 (0.74-0.85)	**x^2 p<.0001**	2.32 (1.59-3.39)	0.93 (0.90-0.96)	**x^2 p <.0001**	3.96 (2.12-7.45)	0.95 (0.93-0.98)	**x^2 p <.0001**
Chlamydia T (+) n (%)	35 (2.55)	18 (51.43)	17 (48.57)	**3.19 (1.62-6.26) (<0.05)**	14 (40.00)	21 (60.00)	**2.53 (1.27-5.03) (<0.05)**	8 (22.86)	27 (77.14)	**4.01 (1.77-9.07) (<0.05)**	4 (11.43)	31 (88.57)	**4.41 (1.48-13.11) (<0.05)**
Chlamydia T (-) n (%)	1336 (97.45)	333 (24.93)	1003 (75.07)		279 (20.88)	1057 (79.12)		92 (6.89)	1244 (93.11)		38 (2.84)	1298 (97.16)	
	RR	2.06 (1.48-2.88)	0.65 (0.46-0.91)	**x^2 p=0.0005**	1.92 (1.26-2.91)	0.76 (0.58-0.10)	**x^2 p<0.01**	3.32 (1.75-6.29)	0.83 (0.69-0.99)	**x^2 p<0.0005**	4.02 (1.52-10.64)	0.91 (0.81-1.03)	**x^2 p<0.005**
Mycoplasma H (+) n (%)	99 (7.22)	42 (42.42)	57 (57.58)	**2.30 (1.51-3.49) (<0.05)**	33 (33.33)	66 (66.67)	**1.95 (1.25-3.02) (<0.05)**	15 (15.15)	84 (84.85)	**2.49 (1.38-4.51) (p<0.05)**	6 (6.06)	93 (93.94)	2.22 (0.91-5.39) (p>0.05)
Mycoplasma H (-) n (%)	1272 (92.78)	309 (24.29)	963 (70.24)		260 (20.44)	1012 (79.56)		85 (6.68)	1187 (93.32)		36 (2.83)	1236 (97.17)	

	RR	1.75 (1.36-2.24)	0.76 (0.64-0.90)	**x^2 p <.0001**	1.63 (1.21-2.20)	0.84 (0.73-0.97)	**x^2 p<0.005**	2.27 (1.36-3.77)	0.91 (0.84-0.99)	**x^2 p<0.005**	2.14 (0.92-4.96)	0.97 (0.92-1.02)	x-square p=0.0724
Mycoplasma G (+) n (%)	11 (0.8%)	3 (27.27)	8 (72.73)	1.09 (0.29-4.13) (p>0.05)	3 (27.27)	8 (72.73)	1.38 (0.36-5.25) (p>0.05)	0 (0)	11 (100)	NA	0 (0)	11 (100)	NA
Mycoplasma G (-) n (%)	1360 (99.2)	348 (25.59)	1012 (74.41)		290 (21.32)	1070 (78.68)		100 (7.35)	1260 (92.65)		42 (3.09)	1318 (96.91)	
	RR	1.07 (0.40-2.81)	0.98 (0.68-1.41)	x^2 p=0.8985	1.28 (0.48-3.38)	0.92 (0.64-1.33)	x^2 p=0.6317	NA	1.08 (1.06-1.10)	x^2 p=0.3503	NA	1.03 (1.02-1.04)	x^2 p=0.5539
Ureaplasma spp (+) n (%)	350 (25.23)	135 (38.57)	215 (61.43)	**2.34 (1.80-3.04) (p<0.05)**	117 (33.43)	233 (66.57)	**2.41 (1.83-3.17) (p<0.05)**	39 (11.14)	311 (88.86)	**1.97 (1.29-3.01) (p<0.05)**	21 (6.00)	329 (94.00)	**3.04 (1.64-5.64) (p<0.05)**
Ureaplasma spp (-) n (%)	1021 (74.47)	216 (21.16)	805 (78.84)		176 (17.24)	845 (82.76)		61 (5.97)	960 (94.03)		21 (2.06)	1000 (97.94)	
	RR	1.82 (1.53-2.18)	0.78 (0.71-0.85)	**x^2 p <.0001**	1.94 (1.59-2.37)	0.80 (0.74-0.87)	**x^2 p <.0001**	1.87 (1.27-2.74)	0.95 (0.91-0.98)	**x^2 p<0.005**	2.92 (1.61-5.28)	0.96 (0.93-0.99)	**x^2 p<0.0005**

RR: Relative Risk, bold values indicate statistical significance (p<0.05) for correlation of specific STD with HPV infection status.
